# Poisoning by Datura Stramonium

**Published:** 1852-03

**Authors:** 

**Affiliations:** Doncaster


*Poisoning by Datura Stramonium.*—By Dr. Breweston of Doncaster.—Early in the afternoon of the 14th of October, I was summoned to see two children of the respective ages of five and three years, who were described by the messenger as having suddenly "gone out of their heads." They had been picked up in the street in this state, and carried into the house of the stranger.

I immediately recognized the symptoms of poisoning by one of the *Solaneæ.* The face was flushed; the eyes were staring and brilliant, and the pupil was fixed and excessively dilated. There was complete loss of vision, imperfect control over voluntary muscular action, and violent delirium, which was alternately mirthful, furious, or dolorous, directed to some imaginary source of pleasure, or repugnance, and accompanied with corresponding gesticulations. The tongue was occasionally thrust from the mouth, and the throat, which appeared to be the only sensible seat of discomfort, was grasped and torn by the hands, as if the patients imagined it to be oppressed by some foreign body. Their attention could not be aroused to anything which was said, and they resisted all attempts at interference with piteous cries or violent struggling.

I suspected the poison of belladonna, and caused search to be made in the neighborhood for the berries of that plant, administering emetic doses of sulphate of zinc in the meantime. The patients exhibited apparent reluctance to swallow, keeping the jaws rigidly closed, and resisting the administration of the remedy with their hands, though they were not sufficiently conscious properly to direct such efforts. The stomach was slow to act, but after considerable perseverance vomiting was induced. Numerous seeds of *datura* were detected in the ejecta; indeed a much greater quantity than has been known to cause death.

It seemed, from the search which had been made, that the poisonous herb had been thrown out from a neighboring garden, and several emptied and divided thorn-apples were discovered. A third child confessed to have eaten a smaller quantity of the seeds, and she was slightly though not alarmingly affected. The poison having been retained four or five hours when vomiting took place, a sharp aperient dose was administered, which, after some hours' delay, acted moderately. Cold was from the first assiduously applied to the head.

At five p. m. four hours from my being first called, though the symptoms were somewhat abated, vision and consciousness to external things were not restored, and the delirium returned at intervals with nearly all its previous violence. The parents of the children, who were now present, were not noticed by them, save occasionally to be embraced or attacked for some imaginary benefit or injury. I subjected the patients to a shower-bath, and applied a blister to the back of the neck.

At nine vision was partially restored in the elder child, and the *hallucinations* were in a great measure supplanted by *illusions,* which indicated some return of consciousness.

At eleven the delirium had become of a muttering type; the patients were drowsy, and displayed a comatose tendency. In the elder child *the pupil, though contracted in the dark, gradually dilated to the stimulus of light.* Sinapisms were now applied to the feet, calves, &c. Alternate doses of strong tea and ammonia were given; the face was occasionally sprinkled with water, and other means were used to prevent sleep.

At five o'clock the ensuing morning, the victims were allowed to sleep, and both awoke before noon, perfectly conscious and well.

On the morning of the third day, the body of the younger child became covered with a small papular eruption, which continued for several days, accompanied with fever.

The effects of the poison were *purely nervous*. The *cerebrum* was first affected; hence arose perverted vision, illusive delirium, and, as consciousness diminished, pure hallucinations. The derangement next attacked the *cerebellum*, as was shown in the loss of control over voluntary muscular action.

Finally, the *medulla spinalis* was implicated, as was evinced by the spasmodic motions of the extremities, &c. In each organ, the first effect seemed to be *stimulation*. This was followed by *perversion*, and lastly, by *depression*, and threatened *extinction* of the function.

It is then incorrect, with such evidence as the above recorded symptoms present, to class *datura* among the *narcotico-irritant* poisons. This term has been vaguely applied by toxicologists to several poisons which are in no proper sense of the term irritants; and which only differ from the *narcotics proper*, either in affecting more peculiarly the spinal cord, or in the stimulating effects—the first result of all narcotics—being more marked or of longer continuance than the narcotism which follows. Death may even occur from over stimulation, before the depression sets in. I have, for instance, poisoned animals with Indian hemp, some of which have died in a state approaching to *tetanus*, while others have survived this state to perish by *coma*, or, at least, by *nervous depression*. It is probable that a poisonous dose of *strychnine*, if it did not first kill by over-excitation of the spinal cord, would eventually do so, like *aconite*, by depression of that organ.—*Prov. Jour. from Dublin Med. Press.*

